# Natural product-induced apoptosis in oral squamous cell carcinoma via targeting mitochondrial multifunctionality

**DOI:** 10.3389/fonc.2026.1749700

**Published:** 2026-03-26

**Authors:** Qi Zhou, Ziyi Wu, Muni Chen, Hao He, Panpan Liu, Mengting Xu, Qianrong Xu, Jiayu Yan

**Affiliations:** 1 School of Clinical Medicine, Chengdu University of Traditional Chinese Medicine, Chengdu, China; 2 Department of Stomatology, Sichuan Integrated Traditional and Western Medicine Hospital, Chengdu, China; 3 Chengdu University of Traditional Chinese Medicine Affiliated Hospital, Chengdu, Sichuan, China

**Keywords:** mechanism of action, mitochondria, natural products, OSCC, targeted therapy

## Abstract

**Background:**

Mitochondria, as the regulatory center of cellular energy metabolism, can influence ATP synthesis, redox balance, and apoptotic pathways. Recent studies have confirmed that some natural products, due to their self-assembling properties and multi-target effects, can inhibit OSCC progression by regulating mitochondrial function, providing key lead compounds for the development of new OSCC therapies.

**Methods:**

We retrieved data from PubMed, Embase, Web of Science, and CNKI to classify and summarize the relationship between mitochondria and OSCC, as well as the regulatory mechanisms of natural products.

**Results:**

Natural products exhibit unique advantages in inducing apoptosis in OSCC cells through multi-pathway regulation mechanisms targeting mitochondria. Various active components, such as betaine and Arglabin, induce oxidative stress by promoting ROS accumulation, disrupting mitochondrial redox balance, and thereby activating the intrinsic apoptotic pathway; extracts like cantharidin and berberine induce changes in mitochondrial membrane permeability by regulating the expression ratio of Bcl-2 family proteins, promoting the release of Cyt-c and activating the caspase cascade. It is noteworthy that combining radiotherapy drugs with natural products for OSCC treatment offers greater stability without additive side effects. Moreover, nanodelivery technology, through the design of smart responsive carriers, introduces a competitive release mechanism, achieving precise, on-demand drug release at tumor sites, providing important candidates for the development of new antitumor drugs.

**Conclusion:**

Natural products targeting mitochondria provide a new direction for the treatment of OSCC, and their potential for clinical translation is expected to overcome existing therapeutic challenges.

## 1 Clinical status of onset carcinogenesis rate of OSCC

OSCC is the most common malignancy of the head and neck. Ulcers are typical of OSCC and are characterized by irregularities at the base and margins that are difficult to distinguish from benign lesions on palpation (1). The posterior lateral edge of the tongue has the highest incidence of OSCC, accounting for about 50% of all OSCC cases (2), followed by the floor of the mouth, soft palate, gingiva, buccal mucosa, and hard palate (3). According to data collected by the Global Cancer Observatory (GCO), there were 389846 cases of OSCC worldwide in 2022, compared to 377,713 cases in 2020, with an annual increase of 1.6% and a mortality rate of 48%. In most cases, surgery is the first-line treatment for OSCC, but inadequate tumor cell removal increases the likelihood of local and regional recurrence and reduces long-term survival; The antibody cetuximab is used in clinical practice for the treatment of OSCC, but its clinical efficacy is limited by side effects, recurrence rate, and drug resistance (4). Therefore, there is an urgent need to develop more effective, non-invasive, and low-toxicity treatment strategies.

Mitochondria are key organelles responsible for energy production within the cell, producing adenosine triphosphate (ATP) through oxidative phosphorylation, and are involved in processes such as metabolic regulation, calcium homeostasis, and apoptosis, and contain an independent genome (mtDNA) (5). An increasing number of studies indicate that mitochondria play a key and dynamic role in the metabolic reprogramming of OSCC; it is not simply a matter of functional inactivation, but rather drives tumor malignancy in synergy with vigorous glycolysis through functional remodeling (6). OSCC cells exhibit a pronounced Warburg effect, rapidly generating energy through aerobic glycolysis and producing large amounts of lactate. This process is precisely regulated by multiple signaling pathways such as ITGB2/PI3K/AKT/mTOR, as well as molecules like PDIA6 and non-coding RNAs. Against this backdrop, the core function of mitochondria shifts from efficient ATP production to supporting biosynthesis (7). The TCA cycle provides essential precursors for nucleic acid, protein, and lipid synthesis and generates metabolic intermediates required for maintaining redox balance and influencing epigenetic modifications. This metabolic reprogramming directly promotes OSCC proliferation, invasion, EMT, and metastasis. Additionally, within the tumor microenvironment, cancer cells interact metabolically with CAFs, immune cells, and others to collectively shape an immunosuppressive environment: CAF-mediated ‘reverse Warburg effect’ enhances glycolysis to ‘fuel’ cancer cells, while cancer cells compete with CD8^+^ T cells for glucose, leading to T cell exhaustion (7). At the same time, the mitochondrial metabolism and function of cancer cells and immune cells like TAMs undergo adaptive changes; for example, impaired mitochondrial function in CD8^+^ T cells weakens their antitumor immunity (8).

Natural medicine (9)refers to chemical substances that are made up of natural substances such as plants, animals, aquatic organisms, or minerals that have pharmacological or biological properties. Due to its structural diversity and multi-target action characteristics, natural product extracts have shown unique advantages in the field of anti-tumor (10), which can effectively inhibit the proliferation, migration and invasion of tumor cells through multi-pathway synergy, and regulate the apoptosis process. Many anti-cancer drugs are developed from natural products and their derivatives, such as paclitaxel (11) and vinblastine (12), which have been clinically validated and exhibit high efficacy with low toxicity.

Studies have shown that natural products not only possess intrinsic anti-tumor activity, but their unique physicochemical properties may also play a role in regulating biological effects. Under certain conditions, natural substances can spontaneously form supramolecular assemblies such as nanoparticles, gels, or micelles through non-covalent interactions like hydrogen bonding, hydrophobic interactions, and π-π stacking. This self-assembly property not only makes them low-toxicity, biocompatible drug delivery carriers, but may also partly explain the material basis and intrinsic mechanisms of their multi-target synergistic effects (13). It is noteworthy that in cancer treatment, chemotherapy often leads to drug resistance due to ‘competitive release.’ After sensitive cell clones are eliminated by the drug, ecological niches are vacated for the original resistant clones, allowing their rapid expansion (14). The self-assembly nature of natural products enhances drug solubility, drug loading, and competitive release, offering new strategies to overcome drug resistance (15).

Natural products combined with conventional radiotherapy or chemotherapy have shown good tolerance and synergistic effects. For example, quercetin can enhance cisplatin-induced intrinsic and extrinsic apoptosis by inhibiting the Akt/IKKβ/NF-κB signaling pathway, downregulating xIAP expression, and activating caspase-8 and caspase-9, thereby synergistically inhibiting OSCC growth both *in vitro* and *in vivo (*16). Neoadjuvant chemotherapy with paclitaxel combined with carboplatin has also achieved an overall response rate of 43% in clinical practice, without significantly increasing side effects based on mechanistic synergy (17). Additionally, the structural optimization of natural product lead compounds and the application of green nano-delivery technologies have further enhanced their antitumor potential and selectivity. Based on this, the present study focuses on natural products targeting mitochondria in OSCC, exploring their molecular mechanisms in treating OSCC, emphasizing the unique mitochondrial damage characteristics of OSCC, and combining nano-drug delivery strategies with the self-assembly properties of natural products to provide new insights for the clinical translation of highly effective and low-toxicity targeted therapies against OSCC.

## 2 The role of mitochondria in OSCC: mechanistic perspectives and diagrams

### 2.1 Mitochondrial metabolic abnormalities amplify cancer cell metabolism through the Warburg effect

The Warburg effect is a specific type of metabolic reprogramming. OSCC depends on the Warburg effect, which is characterized by the preference to rapidly break down glucose through glycolysis and produce a large amount of lactate even under oxygen-rich conditions (18). This metabolic pathway not only provides energy and biosynthetic materials for the rapid proliferation of OSCC cells but also acidifies the tumor microenvironment with accumulated lactate, thereby promoting tumor invasion, metastasis, and inducing immune evasion (19). In this process, mitochondrial reprogramming works synergistically with the Warburg effect, and signals generated by mitochondria can, in turn, enhance glycolysis, collectively constructing a metabolic system that supports rapid growth, biosynthesis, and signal transduction in cancer cells. In addition, cancer-associated fibroblasts (CAFs) in the tumor microenvironment can enhance mitochondrial metabolism through mechanisms such as the ‘reverse Warburg effect,’ providing energy substrates for OSCC cells and further reinforcing the metabolic preferences of cancer cells (20). It is worth noting that the metabolism of OSCC is highly heterogeneous, with certain cancer cell subpopulations or microenvironments still depending on or dynamically switching to oxidative phosphorylation to sustain survival, indicating that therapies targeting metabolism must consider this complexity. This metabolic reprogramming not only promotes immune evasion and the formation of a supportive microenvironment but also enhances cancer cells’ metabolic stress response to chemotherapeutic drugs, highlighting the significant potential of targeting metabolic pathways in OSCC treatment (21).

### 2.2 Mitochondrial DNA damage drives OSCC

Mitochondrial DNA (mtDNA) and nuclear DNA damage are often induced by excessive accumulation of ROS, with mutations in key genes (such as TP53, PIK3CA) driving genomic instability and tumor progression (22). In OSCC, high-frequency mutations and copy number variations in the mtDNA D-loop region are closely associated with lymph node metastasis and poor prognosis. Mutations in nuclear genomic genes involved in mtDNA repair (such as POLG, TFAM) can further impair mitochondrial function, creating a vicious cycle of ‘ROS-mtDNA damage-metabolic disorder.’ This cycle not only leads to ETC dysfunction but also causes the accumulation of cancer metabolites and activation of pro-metastatic signals, directly driving OSCC progression. Research shows that reduced mtDNA content is often accompanied by metabolic reprogramming—sustaining energy supply through suppression of OXPHOS and enhancement of glycolysis (Warburg effect) (22). This adaptive change directly leads to OSCC’s resistance to chemotherapeutic drugs like cisplatin by enhancing antioxidant defenses and acidifying the microenvironment. Therefore, targeting mtDNA or using it as a liquid biopsy marker provides new directions for precision treatment and diagnosis of OSCC.

### 2.3 The dual role of mitochondrial ROS

ROS is mainly produced in mitochondria, which are also the main targets of ROS. Physiological levels of ROS are involved in normal cell signaling (23), but when it accumulates excessively, it triggers a series of pathological changes. High concentration of ROS will promote Ca²^+^ influx, and the increase of intracellular Ca²^+^ concentration will further stimulate ROS production, forming a vicious circle. The synergistic accumulation of ROS and Ca²^+^ acts on the mitochondrial permeability transition pore (mPTP), resulting in a decrease in mitochondrial membrane potential and an increase in permeability (24), which promotes the release of Cyt-c from the mitochondria to the cytoplasm, jointly promotes tumor progression, and mediates chemotherapy resistance.

### 2.4 Caspase activation chain of the mitochondrial pathway

Caspases are a family of proteases that play a key role in apoptosis and inflammatory responses. In OSCC (oral squamous cell carcinoma), the mitochondrial pathway of caspase activation is central to the mechanism of programmed cell death, but its function is often compromised (25). The caspase-9/caspase-3 cascade activation triggered by the release of pro-apoptotic factors (such as Cyt-c) is the execution core of apoptosis. However, OSCC cells escape this pathway through various strategies such as overexpression of anti-apoptotic proteins (Bcl-2, Mcl-1) and frequent mutations in TP53, leading to chemotherapy resistance and poor prognosis (26). As a critical executor, caspase-3 determines cell fate through dual regulation of apoptosis and necroptosis: effective activation can induce apoptosis; when apoptosis is suppressed, its moderate activation may switch to cleaving GSDME, which mediates inflammatory necroptosis, providing new ideas for OSCC treatment.

### 2.5 Mitochondria-mediated resistance to apoptosis

In OSCC, mitochondrial-mediated apoptosis resistance is a key pathological feature, primarily involving the abnormal expression of anti-apoptotic proteins and the disruption of mitochondrial membrane potential (ΔΨm) homeostasis. Overexpression of anti-apoptotic proteins is one of the core mechanisms by which tumor cells evade apoptosis, with the dysregulation of the BCL-2 protein family being particularly critical (27). This family includes three functional subgroups: pro-survival (such as BCL-2, BCL-xL), pro-apoptotic (such as BAX, BAK), and regulatory proteins, which control the permeability of the mitochondrial outer membrane through dynamic balance. In OSCC, overexpression of pro-survival members or reduction of pro-apoptotic proteins can stabilize the mitochondrial outer membrane, inhibit the release of Cyt-c, thereby blocking the activation of downstream caspase cascades, ultimately leading to apoptosis evasion (28). At the same time, OSCC cells often exhibit abnormal elevation of mitochondrial membrane potential (ΔΨm), with molecular bases including ion transport imbalance, altered activity of respiratory chain complexes, or increased stability of the mitochondrial permeability transition pore (mPTP). High ΔΨm inhibits apoptosis through both physical blockade and signal activation mechanisms. It can directly obstruct Cyt-c release channels by affecting functional proteins such as voltage-dependent anion channels (VDAC) and adenine nucleotide translocase (ANT); high ΔΨm can also drive the production of mitochondrial reactive oxygen species (mtROS), activating pro-survival signaling pathways such as NF-κB, which in turn upregulates the expression of anti-apoptotic proteins like Bcl-2, forming a positive feedback loop (28). These changes not only reduce cellular apoptosis sensitivity but are often accompanied by enhanced stemness, increased invasiveness, and intensified therapeutic resistance.

### 2.6 Mitochondria-endoplasmic reticulum interactions and calcium signaling

The endoplasmic reticulum (ER)-mitochondria axis mediates dynamic interactions through mitochondria-associated ER membranes (MAMs), coordinating cellular functions. In OSCC, the dysregulation of MAM-associated protein expression enhances ER-mitochondria coupling, forming efficient Ca²^+^ transport microdomains (29). The ER serves as the primary Ca²^+^ reservoir, releasing Ca²^+^ through channels such as IP3R, while mitochondria efficiently uptake via the MCU protein. This process is precisely regulated by IP3R subtypes, phosphorylation, and interactions with proteins such as Bcl-2, and its homeostatic imbalance presents dual pathological effects: moderate, oscillatory Ca²^+^ signals inhibit apoptosis by activating survival pathways like NF-κB and enhancing mitochondrial metabolism (30), while also increasing tumor migration and invasion by activating calpain proteases; conversely, persistent Ca²^+^ overload caused by MAM dysfunction can induce the opening of the mitochondrial permeability transition pore (mPTP) and functional impairment, which can kill cells (31). However, OSCC evades this pathway by upregulating buffering proteins and anti-apoptotic factors, leading to abnormal cell death and chemotherapy resistance.

**Figure 1:**
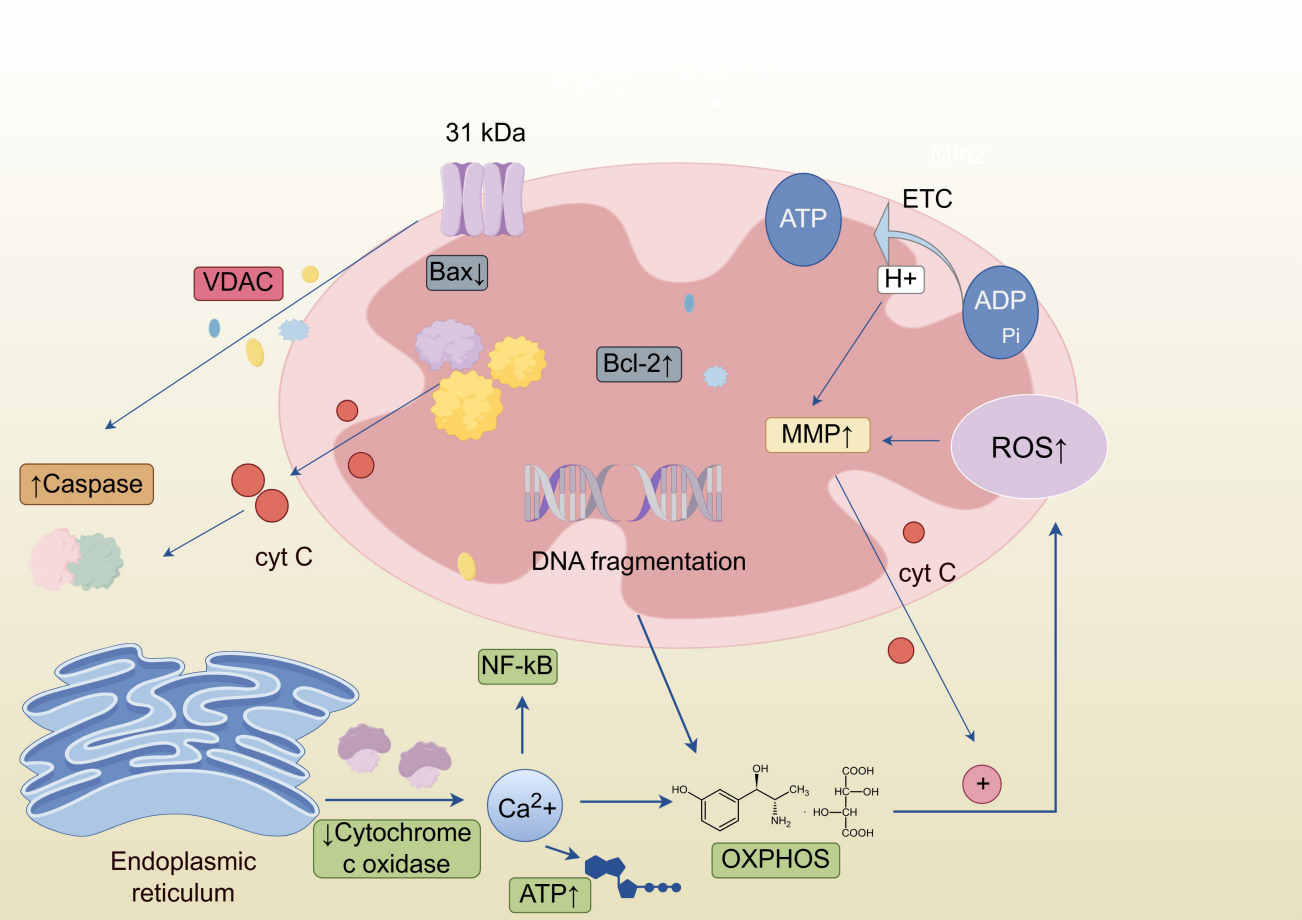
Schematic diagram of mitochondrial mechanisms in OSCC: Abnormal elevation of mitochondrial membrane potential, overexpression of the anti-apoptotic protein Bcl-2, and decreased expression of the pro-apoptotic protein Bax jointly inhibit the release of Cyt-c, blocking the Caspase apoptosis pathway. Meanwhile, excessive ROS generated by mitochondria and abnormal Ca²^+^ release from the endoplasmic reticulum form a vicious cycle, which not only damages mitochondrial DNA and triggers metabolic disorders but also activates pro-survival signaling pathways such as NF-κB, further enhancing apoptosis resistance, collectively promoting the proliferation and invasion of OSCC.

Based on the multidimensional regulatory mechanisms of mitochondrial function described above, we have created a schematic diagram showing how mitochondrial function influences OSCC progression (**Figure 1**), visually presenting the complex regulatory network in which mitochondria serve as a central hub for the malignant progression of OSCC, providing a visual framework for understanding how natural products target mitochondrial multifunctionality to induce OSCC-dependent apoptosis.

## 3 Mitochondria-targeting natural products are used in OSCC treatment

**Figure 2:**
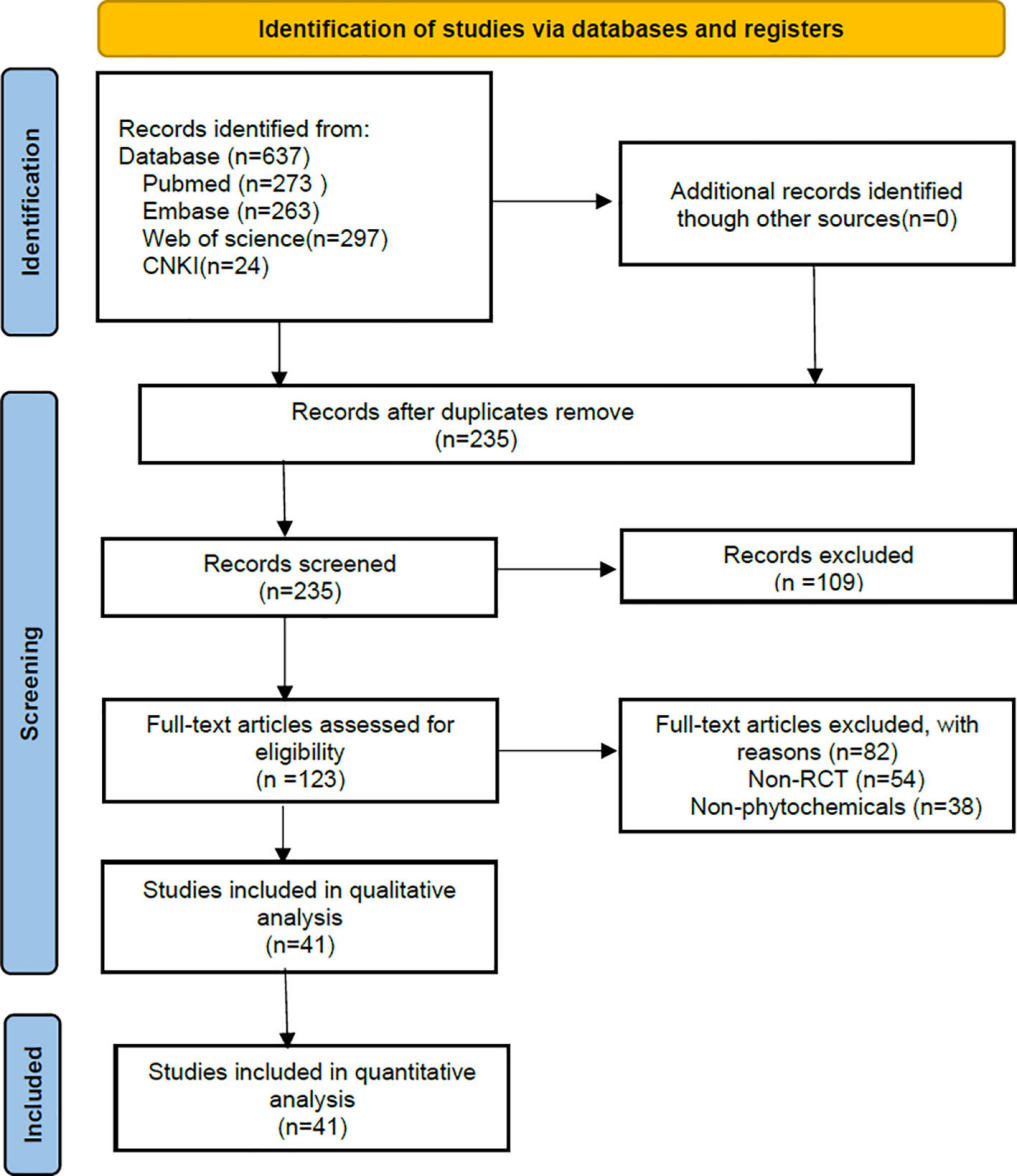
Flowchart of study selection.

Retrieval process - We retrieved 637 records from the initial database search. The keywords used for the literature search included (“Squamous Cell Carcinoma of the Mouth” OR “Oral Cavity Squamous Cell Carcinoma” OR “OSCC” OR “Oral Cancer”) AND (“Mitochondrion” OR “Mitochondrial” OR “Chondriosome”) AND (“Natural product” OR “Phytochemical” OR “Herbal medicine” OR “Botanical drug”). After removing duplicates, the titles and abstracts of 123 studies were evaluated. A total of 82 articles were excluded, and the remaining 41 full-text articles were evaluated, as shown in **Figure 2**.

The filtered study validated the ability of natural products to target mitochondria and induce cell death in OSCC. Natural products are divided into different groups based on the pathways they are used to target mitochondria. These categories include targeting 1) mitochondrial oxidative phosphorylation, 2) mitochondrial DNA fragmentation, 3) regulation of intramitochondrial ROS, 4) caspase activation, 5) mitochondria-mediated resistance to apoptosis, 6) mitochondrial membrane potential, and 7) endoplasmic reticulum (ER)-mitochondrial axis and Ca^2+^ signaling. The classification of natural products and their corresponding mechanisms of action are shown in **Table 1**.

**Table 1:** Natural products targeting mitochondria for the treatment of OSCC.

Source	Bioactive ingredients	Cell culture	*In vivo* studies	Signaling pathways	Study the mechanism	References
Oxidative phosphorylation						
Ganoderma lucidum	G.lucidum spore powder (A-GSP)	SCC15,SCC25	500 or 1000 mg/kg	/	ROS↑ Ferroptosis	(32)
Tangerine Peel	Nobiletin	TCA-8113,CAL27	40 mg/kg/d	cAMP/PKA/CREB	Phosphorylated PKA and phosphorylated CREB, Mitochondria↓	(33)
Licorice	Licochalcones (LCD)	HN22,HSC4	20 mg/kg	JAK2/STAT3	Phosphorylation level↓	(34)
DNA						
Canola oil	UBO	CLS-354	/	/	DNA fragmentation, Nuclear shrinkage, G0/G1 cell cycle arrest	(35)
Sponges (Hippospongia sp)	Rhopaloic acid A	Ca9-22,HSC3,SAS	/	JNK/BNIP3/Nix	DNA damage, Mitophagy	(36)
ROS						
Withania somnifera L	Extracts of W. somnifera fruits and roots	HSC2-4	/	/	ROS↑ Inhibition G2/M	(37)
3-kDa milk betaines	γ-butyrobetaine, δ-valerobetaine	Cal 27	/	/	ROS↑ Inhibition G2/M	(38)
Skin mucous secretions of winter flounder	NRC-03	CAL-27,SCC-9	/	MAPK/ERK ,NF-κB	ROS↑ DNA fragmentation	(39)
Hara	GLA	SCC2,CAL27	40 mg/kg	ATF4/CHOP	ROS↑ Redox imbalance	(40)
Artemisia glabella	Arglabin	SCC4	/	mTOR/PI3K/Akt	ROS↑ MMP↓	(41)
Azadirachta indica	Nimbolide	HSC3,SCC4	/	JNK	ROS,Ca²^+^↑ MMP↓	(42)
Sesamol indicum	3,4-methylenedioxyphenol	SCC25	/	/	Bax,Cyt-c↑ Bcl-2↓	(43)
Glycyrrhiza species	Semilicoiso flavone B	SCC9	/	MAPK, Ras/Raf/MEK	ROS↑	(44)
Gingko biloba leaf	Gingko biloba leaf extract	HSC2	/	/	ROS↑	(45)
Skullcap root	Baicalin	CAL27	/	/	ROS↑ MMP↓	(46)
Water lily	Isoliensinine	HSC3-4	/	MAPK	ROS↑	(46)
Caspase activates the chain						
Ficus septica	Extract of F. septica bark	SCC2095	/	Akt/mTOR/NF-κB, MAPK	caspase-8↓caspase-3,9↑ p-p38,ROS↑	(47)
Anthriscus sylvestris L	Deoxypodophyllotoxin	HSC2-3	/	PI3K/AKT p38 MAPK	caspase-3↑, ROS↑	(48)
Cantharis vesicatoria	*Cantharidin*	CAL-27,SCC-4	/	JNK	Bax,Cyt-c↑ Bcl-2↓	(49)
The climbing stem of Cucumic melo	Cucurbitacin E	SAS	/	/	Caspase 3,MMP↓	(50)
Propolis	Chrysin	KB	/	/	caspase-3/7,MMP↓	(51)
Coptis chinensis	Berberine	HN4	/	/	Bax,Cyt-c,caspase-3/9↑ Bcl-2↓	(52)
Cinnamomum verum J. Presl	Cinnamaldehyde, 4 hydroxycinnamic acid, and eugenol	SCC-25	/	/	caspase-3↑	(53)
Kochia scoparia	Effect of *Kochia scoparia* (MEKS)	Ca9-22,HSC4	Inhibits the growth of OSCC and is non-toxic	/	caspase-3/9↑	(54)
BCL-2 protein family						
Kaffir lime leaves	lupeol, Citronellal, Citronellol	SCC15	/	/	Bax,ROS,caspase-3↑ Bcl-2↓ Inhibition G2/M	(55)
Peony	Ethyl acetate extract of peony seed coat	CAL27	/	STAT3/Survivin	Bax↑ Bcl-2↓	(56)
Impatiens balsamina	Methanol Extracts of balsamina(MEIB)	HSC4,OSC-20,	/	(PI3K)/Akt	Bax↑	(57)
Solanum lyratum Thunberg	Extracts of SLEC	HSC3,SAS,CAL-27	/	/	Bcl-2,Bcl-xl,MMP↓ Bax和Bad,ROS,Ca²^+^↑	(58)
Rumex japonicusHoutt	Physcion 8-O-β-glucopyranoside	KB	40 mg/kg	miR-21/PTEN/Akt/GSK3β	Bax,Bax/Bcl-2,Cyt-c↑ Bcl-2↓	(59)
Cimicifuga foetida	Actein	CAL-27,SCC-9	/	Akt/FoxO1	Bax,Cyt-c,caspase-3↑ Bcl-2↓	(60)
Moringa oleifera	3-Hydroxy-β-Ionone	SCC15	/	/	Bax,Cyt-c,caspase-3/6/7↑ Bcl-2↓	(61)
Grape	Resveratrol	HN4,Cal27,HN6	/	/	Bax,Bax/Bcl-2↑ Bcl-2↓	(62)
Mitochondrial membrane potential						
Mangosteen	α-Mangostin	HSC2-4,Ca9-22,SAS	/	/	MMP↓ caspase-3,9↑	(63)
Pterocarpus marsupium	Gold nanoparticles	SCC29b,SSC154,OEC1	/	/	MMP,ATP↓ ROS↑	(64)
Licorice	Glycyrrhizin	Tca8113	/	/	Cyt-c↑	(65)
Eucommia	Coumarin	HSC2	/	JNK	CDK2,Cyclin A2MMP↓ Autophagy↑	(66)
Bitter gourd seeds	α-MMC	HSC3,	/	/	Bim↑ Mcl-1↓	(67)
Endoplasmic reticulum -mitochondrial axis and Ca^2+^ signaling						
Rosemary and salvia	Carnosic acid	CAL27,SCC9	20 mg/kg		ROS,Ca^2+^↑ MMP↓	(68)
Sea bass	Piscidin-1	SCC4	/	/	Bax,ROS,Ca^2+^↑ Bcl-2,MMP↓	(69)
Essential oils of plants	Thymol	Cal27,SCC4,SCC9	4.3 mmol/L	/	Ca^2+^↑ MMP↓	(70)

↑, Promote; ↓ Inhibit.

### 3.1 Targeting mitochondrial phosphorylation of natural products for the treatment of OSCC

Natural products targeting mitochondrial oxidative phosphorylation can synergistically intervene in mitochondrial oxidative phosphorylation function from three different levels: energy metabolism, mitochondrial biogenesis signaling, and transcriptional regulatory networks, ultimately inducing mitochondrial pathway-mediated OSCC cell death. Ganoderma lucidum spore powder (A-GSP) (32) directly disrupts mitochondrial cristae structure, inhibits ATP production, and interferes with the cystine/glutamate transport system, thereby triggering ferroptosis and mitochondrial membrane hyperpolarization, ultimately leading to apoptosis. Nobiletin (33) extracted from dried tangerine peel acts on the cAMP/PKA/CREB signaling axis, inhibiting the pathway by reducing the levels of phosphorylated PKA and CREB, which impairs mitochondrial biogenesis and function. Licorice-derived chalcone compound Licochalcones (34) directly binds to and inhibits JAK2, blocking downstream STAT3 activation, thereby downregulating a series of STAT3-regulated pro-survival proteins and reshaping the mitochondrial apoptosis threshold.

### 3.2 Natural products targeting mitochondrial DNA for the treatment of OSCC

Natural products targeting mitochondrial DNA trigger a series of downstream cell death events by directly or indirectly damaging the genetic integrity of cancer cells. Canola oil extract (UBO) (35) primarily causes oxidative damage to nuclear DNA, leading to changes in nuclear morphology, G0/G1 cell cycle arrest, and DNA fragmentation, while activating autophagy; this effect is independent of the typical caspase 3/7 apoptosis pathway, demonstrating a unique anti-tumor mechanism. In contrast, the sponge-derived terpene compound Rhopaloic acid A (RA) (36) can induce DNA damage and activate caspase-3-mediated mitochondrial apoptosis. Additionally, RA activates BNIP3/Nix-mediated mitophagy by upregulating JNK and inhibiting the Akt/mTOR/p70S6K signaling pathway, resulting in mitochondrial membrane potential collapse and cell death. This multi-functional mechanism has been confirmed to effectively inhibit OSCC growth in zebrafish models.

### 3.3 Natural products that target ROS in OSCC

In OSCC treatment, natural products targeting mitochondrial ROS constitute one of the most abundant strategies, with their core mechanism being the induction of abnormal mitochondrial ROS accumulation, leading to lethal oxidative stress. These compounds come from diverse sources and have various mechanisms, but they ultimately lead to apoptosis and cell cycle arrest. For example, Withania somnifera L extracts (37) and 3-kDa milk betaines-derived betaines (δVB/γBB) (38) directly cause G2/M phase arrest by inducing ROS bursts. The bioactive peptide NRC-03 (39) significantly promotes ROS generation by specifically upregulating the activity of mitochondrial respiratory chain complex I, subsequently activating the MAPK/ERK and NF-κB pathways and opening the mitochondrial permeability transition pore (mPTP), resulting in energy metabolism collapse. Several plant-derived components amplify the toxic effects of ROS by inhibiting key survival signaling pathways, such as Arglabin (41) and Semisynthetic Isoflavone B (SFB) (44), which act by downregulating the mTOR/PI3K/Akt and Ras/Raf/MEK/MAPK pathways, respectively; while compounds like Water lily (42) and Gingko biloba leaf extracts (40) mainly activate the ROS-MAPK axis. Moreover, some compounds can integrate stress signals from multiple organelles: Nimbolide (42) and Gentian Kaempferol A (GLA) (40), while inducing ROS, respectively activate ER stress-related JNK and ATF4/CHOP pathways, synergizing with the mitochondrial apoptosis pathway. Ultimately, ROS accumulation directly disrupts mitochondrial membrane potential (46) (as seen with Baicalein) and modulates the balance of Bcl-2 family proteins (43) (as with Sesamol), leading to Cyt-c release and activation of the caspase cascade, thus irreversibly executing the apoptosis program.

### 3.4 Natural products targeting the caspase activation chain of the mitochondrial pathway for the treatment of OSCC

Natural products targeting the mitochondrial pathway Caspase activation cascade are core executors of OSCC apoptosis. They precisely trigger programmed cell death by directly or indirectly regulating the Caspase cascade. Among them, classic activators of the intrinsic apoptosis pathway, such as berberine (52) and cantharidin (49), induce collapse of the mitochondrial membrane potential and release of Cyt-c by downregulating Bcl-2 and upregulating Bax, thereby activating the apoptosome and sequentially activating Caspase-9 and Caspase-3. Regulators of upstream signaling pathways drive Caspase activation by affecting key survival or stress pathways. Deoxypodophyllotoxin(DPT) (48) and extract of F. septica bark (FSB) (47) promote Caspase-3 activation by inhibiting the PI3K/AKT pathway and modulating the Akt/mTOR/NF-κB and MAPK pathways (enhancing p38, inhibiting JNK/ERK), respectively. The pro-apoptotic effect of Kochia scoparia extract (MEKS) (54) depends on the activation of p38 MAPK and has been validated in animal models. Notably, some components exhibit unique modes of action: Chrysin (51) and Cinnamomu (53) extract significantly decrease mitochondrial membrane potential while activating Caspases; meanwhile, cucurbitacin E (CuE) (50) shows a unique inhibitory effect on Caspase-3 activity, suggesting that it may act through a non-classical pathway.

### 3.5 Natural products targeting the BCL-2 protein family for the treatment of OSCC

Targeting mitochondrial-mediated apoptosis resistance is a key strategy to overcome the therapeutic challenges of OSCC. Such natural products mainly reactivate the intrinsic apoptotic program in cancer cells by regulating the balance of Bcl-2 family proteins. Natural products can directly modulate the expression and function of Bcl-2 family proteins. For instance, Kaffir lime leaves extract and its active components (55) can significantly induce ROS generation, upregulate the pro-apoptotic protein Bax, and inhibit the anti-apoptotic protein Bcl-2. Extracts of Solanum lyratum (SLEC) (58), Physcion 8-O-β-glucopyranoside (PG) (59), the Moringa active component 3-HBI (61), and resveratrol (62) can also initiate mitochondrial apoptosis by increasing the Bax/Bcl-2 ratio. Additionally, interventions targeting upstream key survival signaling pathways further enhance pro-apoptotic effects. For example, extracts of Impatiens Balsamina (57) activate Bax by inhibiting PI3K/Akt signaling, actein from Cimicifuga foetida (60) downregulates Bcl-2 and upregulates Bim by blocking the Akt/FoxO1 axis, and peony seed coat extract (56) inhibits miR-424-3p, thereby blocking the downstream STAT3/Survivin pro-survival pathway. Furthermore, some natural products exhibit multi-mechanistic synergistic effects. For instance, extracts of SLEC (58), while modulating Bcl-2 family proteins, also synergistically induces ROS and Ca²^+^ accumulation and decreases the mitochondrial membrane potential, producing a multi-pathway enhanced pro-apoptotic effect.

### 3.6 Natural products targeting mitochondrial membrane potential for the treatment of OSCC

Natural products that target mitochondrial membrane potential can induce apoptosis in OSCC cells by disrupting the functional homeostasis of this critical organelle. α-Mangostin derived from mangosteen (63) can directly trigger the collapse of mitochondrial membrane potential, leading to Cyt-c release and activation of the classical caspase-9/-3 apoptotic cascade, and can act synergistically with TRAIL. Nanotechnology enhances the efficacy of natural products; for example, gold nanoparticles prepared from Pterocarpus marsupium bark extract (Pm-AuNPs) (64) can disrupt membrane potential through physical interactions, and preliminary toxicity assessments show good biocompatibility. Glycyrrhizic (65) induces the sustained opening of the mitochondrial permeability transition pore (MPTP), dissipating the proton gradient and thereby collapsing the membrane potential. The Eucommia coumarin derivative B-2 (66) not only disrupts membrane potential and activates caspases but also upregulates autophagy, forming a multi-modal anti-tumor effect. α-momorcharin(α-MMC) from bitter melon seeds (67) regulates the interactions of Bcl-2 family proteins by upregulating the pro-apoptotic protein Bim and inhibiting Mcl-1, indirectly leading to mitochondrial outer membrane permeabilization and loss of membrane potential.

### 3.7 Natural products targeting the endoplasmic reticulum (ER)-mitochondrial axis and Ca^2+^ signaling for the treatment of OSCC

Targeting endoplasmic reticulum (ER)-mitochondria coupling and the calcium (Ca²^+^) signaling it mediates is a cutting-edge strategy for inducing apoptosis in OSCC. Such natural products disrupt the dynamic communication and Ca²^+^ homeostasis between the two organelles, synergistically triggering ER stress and mitochondrial dysfunction, thereby effectively disturbing the internal balance of OSCC cells and driving their death. Among them, Caffeic acid (CA) derived from Rosemary and Salvia (68) can simultaneously elevate intracellular ROS and Ca²^+^ levels, directly causing a decrease in mitochondrial membrane potential (MMP) and cristae structure disintegration, thus promoting apoptosis. The antimicrobial peptide Piscidin-1 from bass (69) induces mitochondrial dysfunction by regulating the Bcl-2 family, while also triggering ER stress and activating the UPR pathway, disrupting Ca²^+^ transfer between the ER and mitochondria, forming a ROS-Ca²^+^ positive feedback loop, and enhancing the caspase cascade. The terpenoid compound thymol (70) exhibits a concentration-dependent effect: at low concentrations, it indirectly affects mitochondrial function by inducing ER stress; at high concentrations, it can directly cause mitochondrial depolarization.

## 4 Clinical translational potential and challenges

### 4.1 Combined treatment strategy

#### 4.1.1 Self-assembly of drugs

In the drug treatment of OSCC, the combination of paclitaxel and platinum compounds is the core regimen for locally advanced and recurrent/metastatic disease (11). However, its clinical application still faces challenges such as low solubility, poor targeting, and systemic toxicity. To overcome these limitations, smart delivery systems that combine prodrug strategies with drug self-assembly show significant promise. Prodrugs (71) are compounds with low or no pharmacological activity themselves but can release active drug molecules in the body through specific biotransformations, aiming to improve the selectivity, stability, or pharmacokinetic properties of the parent drug. Endogenous stimulus-responsive prodrugs have been widely used in anti-tumor therapy because of their lesion-specificity and reduced systemic toxicity. Self-assembly (15) (72) refers to the inherent property of drug active ingredients to spontaneously form supramolecular assemblies with defined structural features and stability under the driving influence of specific environmental conditions, through various non-covalent interactions such as hydrogen bonding, electrostatic interactions, hydrophobic effects, π-π stacking, coordination bonds, and van der Waals forces. Jiang S (73) developed a ROS-responsive heterodimeric prodrug NBS-TK-PTX, which co-assembles with glucose oxidase (GOx) to form the NTP@GOx nanocomposite, achieving a self-boosting multimodal synergistic anti-tumor effect. Similarly, OSCC treatment targeting mitochondrial function also has some experimental basis. Zhong J (15) prepared a carrier-free self-assembling prodrug from two triterpenes isolated from medicinal plants, licorice, and ginseng (glycyrrhetinic acid GA and ginsenoside Rh2), for targeted and efficient OSCC therapy. The ROS self-supplying molecule TK-GA2 was synthesized with a ROS-responsive thiophosphate linker, and the prodrug was prepared with TK-GA2 and Rh2 through a rapid solvent exchange method. After administration, oral tumor cells competitively take up large amounts of the glucose-ligand-containing prodrug. The endogenous ROS within oral tumor cells promotes the release of GA and Rh2. GA further induces the production of large amounts of ROS, helping self-enhance drug release and increase oxidative stress, synergizing with Rh2 to trigger tumor cell apoptosis.

#### 4.1.2 Nanodelivery and therapeutic paradigm shift

In recent years, combining natural products with nanodelivery systems has become an important approach to enhance their therapeutic potential for OSCC. These nanoplatforms can not only improve the water solubility and stability of natural products, but also, through targeted design and size characteristics, more effectively address the anatomical and microenvironmental challenges of OSCC. Lan Q (73) developed the GE11-CuS@Gal nanosystem, which can actively target OSCC cells and release the drug galantamine under near-infrared light control, significantly inhibiting tumor growth by increasing ROS and suppressing antioxidant pathways. The team led by Wang Y designed a self-assembling carrier-free nanoparticle GABG, which can efficiently load paclitaxel and, through simulations, confirmed the stability of its structure, thereby enhancing drug accumulation at the tumor site.

Given the tendency for lymph node metastasis in the middle and late stages of OSCC, nanotechnology systems can achieve targeted delivery to lymphatic areas through surface modification, helping to suppress metastatic lesions (74, 75). At the same time, they can serve as carriers for radiosensitizers, increasing the tumor’s sensitivity to radiation. In addressing the issue of chemotherapy resistance, Zhao (76) developed supramolecular nanomedicines capable of co-delivering drugs with different mechanisms, restoring tumor cell sensitivity to cisplatin by blocking specific resistance signaling pathways. Compared to conventional radiotherapy and chemotherapy, natural product-based nanomedicines show better specificity. Traditional chemotherapeutic drugs often cause severe damage to the oral mucosa, whereas targeted natural product systems can achieve more precise effects, reducing toxic side effects. For example, a prodrug nanosystem self-assembled from glycyrrhetinic acid and ginsenoside Rh2 offers a highly effective and low-toxicity new approach for OSCC treatment (77). Additionally, the nanocarriers themselves can enhance the stability and bioavailability of natural compounds such as naringenin (78), allowing them to act more effectively within tumor tissues.

### 4.2 Current status of clinical research

The occurrence of OSCC is a multistep process driven by both the intrinsic characteristics of cancer cells and the TME. Among these, metabolic reprogramming is a key feature. OSCC cells preferentially utilize the Warburg effect (upregulating GLUT, HK, LDHA) to rapidly generate energy and metabolic intermediates even under aerobic conditions, leading to lactate accumulation (21). The TME is both a product and a site of metabolic reprogramming (79), where CAFs act as core regulators, enhancing glycolysis through mechanisms such as ITGB2 and lncRNA H19 to provide energy for cancer cells, while their secreted lactate also promotes stem cell-like properties (76). Meanwhile, immune cells also undergo metabolic reprogramming: enhanced glycolysis in CD4^+^ T cells may promote metastasis, metabolic adaptation of regulatory T cells contributes to immune evasion, and competition between cancer cells and CD8^+^ T cells for glucose leads to CD8^+^ T cell dysfunction (80).

Although preclinical studies, *in vitro* and animal models have fully demonstrated the significant potential of natural products to target mitochondria and induce apoptosis in OSCC, their clinical translation still faces significant challenges, resulting in limited large-scale, confirmatory Phase II, III clinical trial data at present (81, 82). OSCC is often diagnosed at a mid-to-late stage, with standard clinical treatment primarily consisting of surgical resection combined with chemoradiotherapy or targeted/immunotherapy (83). In this context, using a single natural product with complex components and incompletely understood pharmacokinetic properties as a first-line monotherapy lacks feasibility both ethically and in terms of efficacy. Therefore, the clinical positioning of natural products is shifting from the traditional ‘alternative therapy’ to a more strategically valuable role as ‘adjuvant sensitizers,’ primarily reflected in their combined use with standard treatments. At the same time, natural products can also serve as starting points for innovative drugs, allowing the development of structurally defined, precisely targeted small-molecule drugs based on the inherent self-assembly properties of components such as triterpenes, polyphenols, and saponins.

## 5 Conclusion

In recent years, studies have found that natural products such as terpenoids, alkaloids, saponins, and flavonoids regulate OSCC cell apoptosis through multi-level mechanisms. In addition to directly regulating the Bcl-2/Bax protein balance, inducing mitochondrial membrane potential decline, and ROS bursts, promoting the release of Cyt c and activating the caspase cascade, these compounds also precisely modulate the mitochondrial apoptosis process through upstream signaling networks. Research has shown that natural products can influence the activation and mitochondrial translocation of BH3-only proteins such as BAD, Bim, and Bid by inhibiting the PI3K/Akt/mTOR pathway, regulating phosphorylation of the MAPK family (JNK, p38, ERK), blocking STAT3 transcriptional activity, and affecting the Akt/FoxO1 signaling axis, ultimately controlling mitochondrial outer membrane permeability. In addition, some natural products (such as triterpenes and saponins), due to their inherent chemical amphiphilicity, can self-assemble into carrier-free nanostructures with defined structures. Recent studies have shown that the self-assembly of traditional Chinese medicine not only endows such supramolecular structures with reversibility, dynamic responsiveness, and thermodynamic adaptability, but also fundamentally reshapes the solubility, membrane permeability, and metabolic stability of active components, converting microscopic intermolecular interactions into macroscopic synergistic pharmacological effects. This mechanism provides new strategies for understanding the role of natural products in combination therapies. When synergized with radiotherapy, chemotherapy, or immunotherapy, natural products may exert effects similar to the ‘mutual complementarity, toxicity reduction, and efficacy enhancement’ seen in traditional Chinese medicine compatibility. Self-assembling natural product nanosystems not only significantly improve the solubility and stability of drugs, but also enhance anti-tumor effects by collaboratively triggering multi-level mitochondrial apoptosis signaling pathways, helping to reduce the risk of resistance associated with single-target drugs.

Although natural products targeting mitochondria have shown significant anti-OSCC potential *in vitro*, their clinical translation still faces serious challenges. Current research is mainly limited to cell models, and most *in vitro* evidence relies on extraction and processing with organic solvents (such as methanol and ethyl acetate), whose own potential cytotoxicity may interfere with the accurate assessment of the active components’ true efficacy. Meanwhile, natural products generally have poor pharmacokinetic properties, such as low oral bioavailability, poor metabolic stability, and insufficient tissue selectivity, which restrict their clinical application. Future studies should move beyond *in vitro* mechanistic exploration to validate their potential to modulate the tumor microenvironment and reverse immunosuppression in clinical trials, and, combined with clinical feedback, clarify their specific targets and metabolic fate in the human body, thereby promoting this strategy toward a precise and translatable direction.
